# Case Report: A rare case of antenatally diagnosed mature adrenal teratoma in an infant: insights and literature review

**DOI:** 10.3389/fped.2024.1460251

**Published:** 2024-11-29

**Authors:** Amar Msarweh, Mohammad Hakam Shehadeh, Ahmad M. Abualrub, Waleed M. Malhes, Nadeen Msarweh, Jenan Khaled Sinokrot, Ahmed H. Aliwisat

**Affiliations:** ^1^ Faculty of Medicine, Al-Quds University, Jerusalem, Palestine; ^2^ Department of Pediatric Surgery, Al-Makassed Islamic Charitable Hospital, Jerusalem, Palestine

**Keywords:** teratoma, adrenal gland, mature teratoma, retroperitoneal, prenatal screening

## Abstract

Teratomas are germ cell tumors that arise from the derivatives of the three germ cell layers. They are categorized into subtypes by the extent of maturation, with mature teratomas being the most common subtype. While they can arise in various extragonadal regions, including the retroperitoneum, their occurrence in the retroperitoneal space is extremely rare. It is even more exceptional for these tumors to be located within the adrenal gland. In this report, we describe an 18-day-old female infant who presented with a left suprarenal mass. The mass was detected during prenatal screening via ultrasound at 30 weeks of pregnancy. Evaluation after birth, including a chest and abdomen computed tomography (CT) scan, revealed a large, well-defined left suprarenal mass. The mass was surgically resected and found to measure 9 cm × 7 cm × 5 cm. Histopathological examination confirmed a cystic mature teratoma containing a variety of well-differentiated tissues. The patient has shown excellent progress over the 1-year follow-up, with no evidence of recurrence. Only a few cases of mature adrenal teratoma have been reported, highlighting the importance of this case report.

## Introduction

Retroperitoneal teratomas (RPTs) are the third most prevalent retroperitoneal tumors in the pediatric population, following neuroblastoma and Wilms tumors (1). However, it is important to note that the majority of these lesions are benign in nature. Interestingly, teratomas are exceedingly rare and uncommon tumors, with an estimated incidence rate of only 0.9 per 100,000 in the general population (2, 3). Moreover, primary adrenal teratomas in infants represent an exceptionally rare subset of this already uncommon category. As a result, only a limited number of cases have been documented and published in the medical literature, underscoring the unique and elusive nature of these tumors.

In this report, we present a compelling case of an 18-day-old female infant diagnosed with a mature adrenal teratoma. This case report has been reported in accordance with the SCARE criteria (4).

## Case presentation

An 18-day-old female infant, born to healthy and non-consanguineous parents, was delivered via normal vaginal delivery and had no previous medical history, presented with a left suprarenal mass that was detected during prenatal screening. The mass was initially identified during a routine antenatal ultrasound scan at 30 weeks of pregnancy, which indicated a sizable cystic solid mass in the left adrenal region, raising concerns for potential malignancy. The mass was monitored throughout the remainder of the pregnancy, with subsequent ultrasounds confirming its size and characteristics, showing no significant changes in appearance or maternal health during this period. After birth, ultrasound examination revealed a sizable cystic solid mass in the left adrenal region, measuring approximately 7.9 cm × 4.7 cm. The mass was observed to cause indentation and displacement of the left kidney downwards, while the rest of the examination was unremarkable, except for fullness in the left abdomen.

**Figure 1:**
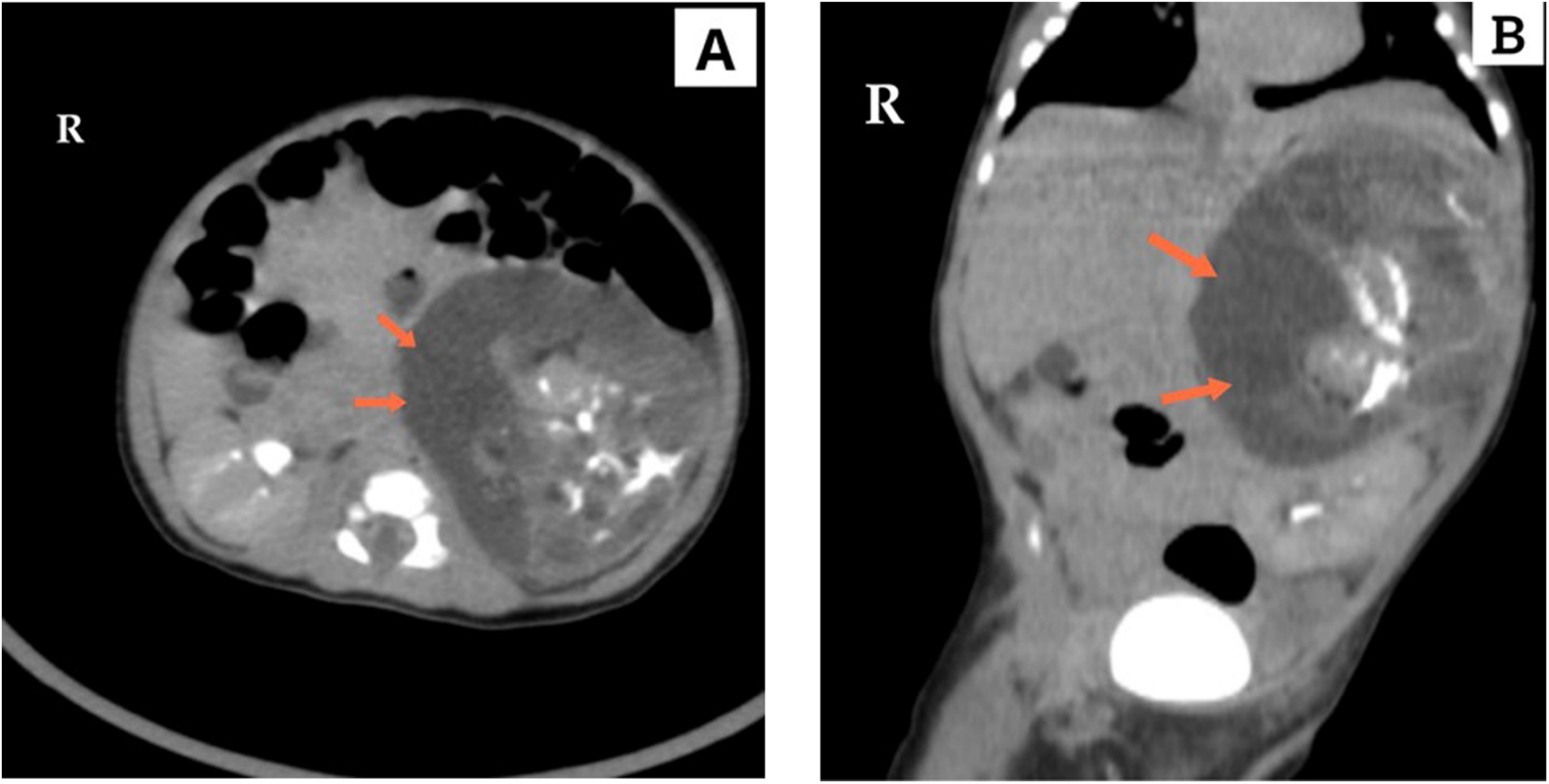
Computed tomography (CT), in **(A)** axial and **(B)** coronal section, demonstrates a giant tumor in the left adrenal region with variable tissue components, including calcification, fat, solid areas, and cystic regions. The tumor's significant size is evident as it displaces the left kidney and adjacent structures.

Routine biological assessments, including liver and kidney function tests as well as a complete blood count, were unremarkable. Additionally, laboratory tests, including tumor markers (α-fetoprotein and β-human chorionic gonadotropin), were all within normal ranges. Further evaluation was conducted through urine gas chromatography-mass spectrometry (GC-MS) analysis, which did not reveal any diagnostic abnormalities, including the absence of homovanillic acid (HVA) or vanillylmandelic acid (VMA) excretion. It is important to note that normal values do not exclude the presence of catecholamine-secreting tumors. Subsequently, a chest and abdominal computed tomography (CT) scan was performed, revealing a large, well-defined left suprarenal mass measuring 8.5 cm × 6.5 cm (Figure 1). The mass displayed a variety of tissue components, including calcifications, fat, solid areas, and cystic regions. The lesion exerted a significant mass effect on the surrounding structures, anteriorly displacing the spleen, splenic vessels, pancreas, and portions of the stomach body. Medially, it pushed against the superior mesenteric artery and inferiorly displaced the kidney to the left iliac fossa, resulting in a transverse kidney position. The renal vein was displaced inferiorly and medially. Both kidneys exhibited normal excretion, and no distant metastases were observed. The mass was determined to be resectable and did not invade any vessels.

**Figure 2:**
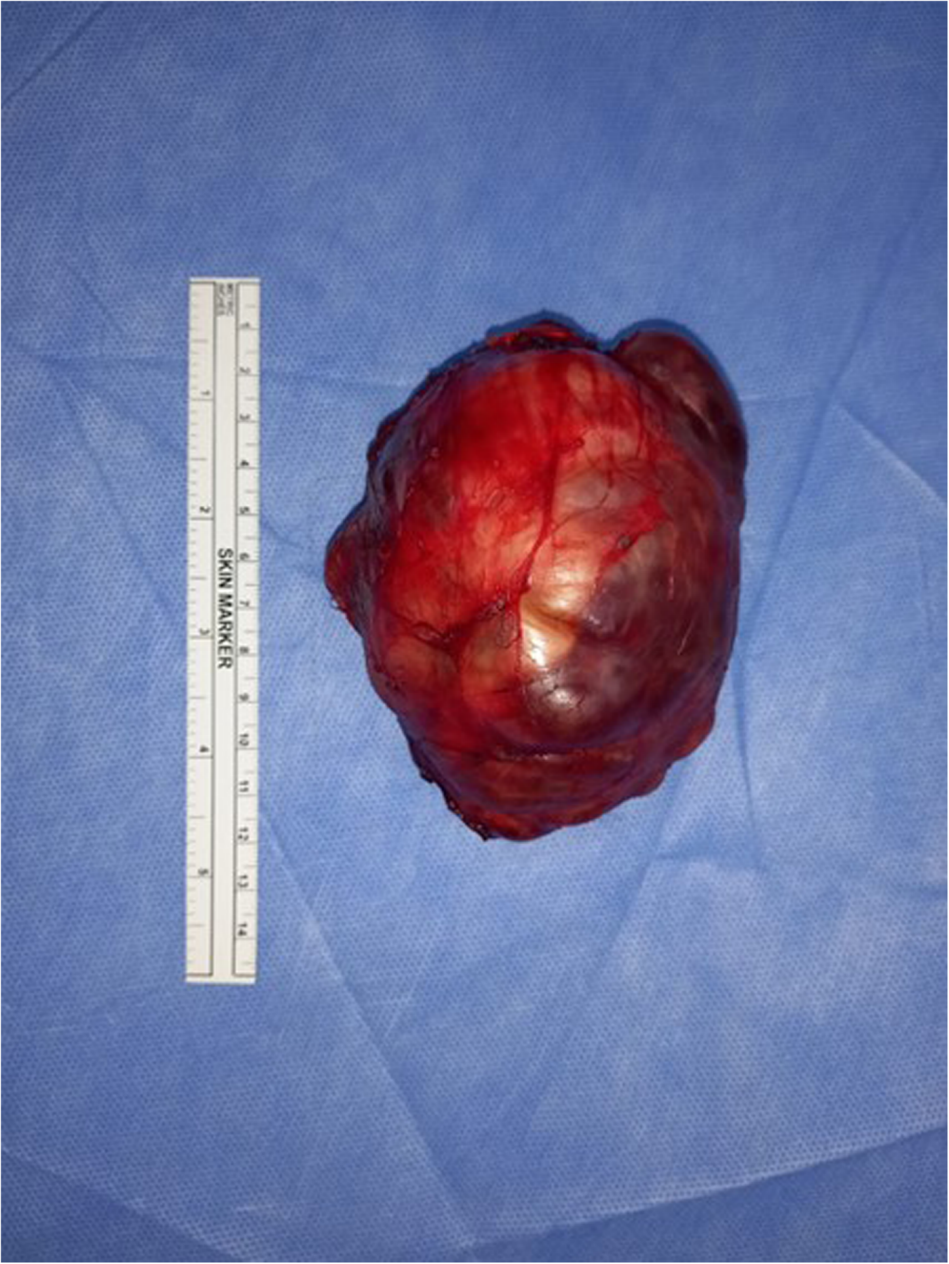
Gross appearance of the restricted tumor showing a large irregular cystic and solid adrenal mass.

To further assess the cardiac status, an echocardiogram was performed, which revealed normal findings. Based on the suspicion of neuroblastoma, surgical resection of the mass was planned. During the procedure, the mass was completely excised with gross dimensions of 9 cm × 7 cm × 5 cm (Figure 2). The tumor had an irregular shape and was surrounded by a thin capsule. On sectioning, solid and cystic components with bone structures were observed. Notably, the mass was distinct from the adjacent normal adrenal tissue, which was significantly displaced but not entirely subverted. A partial adrenalectomy was performed to preserve adrenal function while ensuring complete removal of the tumor.

Histopathological examination revealed a cystic mature teratoma. Immature elements were present in less than one low-power field and accounted for approximately 2% of the extensively sampled tissues. The mature components include a variety of well-differentiated tissues, such as skin, hair follicles, sebaceous glands, cartilage, and bone. The tumor was composed of 98% mature elements.

The patient has shown excellent progress during the 1-year follow-up period, exhibiting no signs of recurrence based on clinical, radiological, and biochemical evaluations.

## Discussion

Teratomas are germ cell tumors that arise from totipotent cells and contain various cell types derived from the three germ layers (ectoderm, mesoderm, and endoderm) (5). During the fourth week of embryonic development, germ cells originating from the yolk sac migrate along the midline of the fetus, specifically through the dorsal mesentery, from the urogenital ridge towards the developing gonads. However, some of these cells fail to complete their migration and instead persist in midline locations such as the pineal gland, anterior mediastinum, retroperitoneum, and sacrococcygeal areas. Germ cells differentiate into extragonadal teratomas in these locations. They can be classified into four types based on their degree of maturation: mature, immature, teratoma with malignant transformation, and monodermal (3, 6). While gonadal teratomas are more common in adults, extragonadal teratomas can occur in various locations such as the mediastinum, retroperitoneum, cranium, sacrococcygeal region, large bowel, and tongue (3).

In pediatric cases, teratomas are commonly found in the sacrococcygeal region (41%), ovaries (28%), and testes (7%) (7). Approximately 24% of teratomas are found in midline structures such as the mediastinum and retroperitoneum. Among these, mediastinal teratomas account for 6%–18% of pediatric mediastinal tumors, with the majority of cases being benign in nature (8). The retroperitoneal location, specifically within the adrenal gland, is extremely rare, comprising only 1%–11% of all primary tumors in the retroperitoneal region (9).

Retroperitoneal mature cystic teratomas exhibit an interesting pattern of incidence, with two distinct peaks observed in the first six months of life and early adulthood (9). Approximately half of all cases are diagnosed within the first year of life, indicating a higher prevalence during infancy (9). Conversely, only 10%–20% of these teratomas occur in adults aged 30 years and older (10). Interestingly, the left suprarenal region, which is the location of the tumor in this patient, appears to be the predominant site for retroperitoneal cystic teratomas (10).

The retroperitoneum provides ample space for these tumors to grow, resulting in a typically substantial size upon presentation. Symptoms may manifest as abdominal or back pain, and in some cases can even cause intestinal obstruction due to compression. Alternatively, some cases may be asymptomatic and may be incidentally discovered. The nonspecific nature of these symptoms presents a challenge for early diagnosis. Although most adrenal teratomas are benign (6), they can give rise to complications. Tumor rupture poses a potential risk, leading to sudden-onset abdominal pain, ascites, hemorrhage, and peritonitis (2, 11, 12).

Diagnosing suprarenal tumors in infants during the antenatal or early neonatal periods poses significant diagnostic challenges. When evaluating a potential adrenal teratoma, it is essential to consider other conditions that may present as suprarenal masses. While neuroblastoma is the most frequently detected suprarenal mass during prenatal ultrasound, it is crucial to include other possibilities, such as adrenal hemorrhage, extrapulmonary sequestration, bronchogenic cyst, ovarian tumors, lymphangioma, and renal dysplasia, in the differential diagnosis (1, 6). Additionally, other adrenal tumors with a fatty component, including myelolipoma, lipoma, angiomyolipoma, and liposarcoma, should be considered when differentiating from adrenal teratomas (13).

The detection of antenatal mature adrenal teratomas is exceedingly rare. These tumors present as complex cystic masses within the fetal abdomen. Diagnostic imaging is crucial for the diagnosis of adrenal teratomas, as laboratory findings are typically nonspecific. Modalities such as plain radiography, abdominal ultrasound, computed tomography (CT), and magnetic resonance imaging (MRI) of the abdomen are useful for diagnosing teratomas (14). Radiological features, such as calcification, teeth, and fat, can help in diagnosis. Ultrasonography is often the initial imaging method used in pediatric cases and can differentiate between cystic and solid components (1). CT and MRI are essential for assessing the extent of the tumor in the retroperitoneum and its relationship to major blood vessels, facilitating preoperative planning, and increasing the likelihood of complete tumor removal with minimal iatrogenic damage (1). Internal homogeneity, fat density, cyst formation, and calcification are significant predictors of benign retroperitoneal teratomas on CT. MRI provides superior resolution for soft tissues, allowing the identification of benign and malignant neoplastic features and facilitating tumor staging assessment (15). Histopathological analysis following laparoscopic adrenal tumor resection is often required for a definitive diagnosis.

Most adrenal teratomas are unilateral, with only one documented case of bilateral involvement (16). In patients with bilateral adrenal tumors requiring surgery, subtotal adrenalectomy may be recommended to avoid permanent adrenal insufficiency and steroid dependency, along with its associated risks, such as decreased quality of life and increased morbidity (17, 18). This technique involves removing adrenal pathology while preserving vascularized adrenal tissue to maintain hormone homeostasis. However, it is infrequently performed, and many clinicians may lack familiarity with its considerations; approximately 30% of normal adrenal tissue is needed to ensure adequate steroid levels (18).

Complete surgical resection followed by close monitoring is recommended for mature teratomas and is necessary for definitive diagnosis (1–3). Laparoscopic surgery has emerged as the primary treatment modality for benign adrenal tumors, surpassing open surgery. This shift is attributed to several advantages associated with laparoscopic approaches, including reduced morbidity, faster recovery, and decreased postoperative pain (19). However, in the case of an immature teratoma, adjuvant therapy, such as chemotherapy, radiotherapy, or concurrent chemoradiotherapy, may be required following complete resection of the primary tumor (2). The prognosis of patients with mature adrenal teratomas is generally favorable following complete surgical resection. Long-term follow-up is recommended to monitor for recurrence or metastasis, although these are uncommon (2). Currently, there is a lack of sensitive indicators for effectively monitoring the relapse of adrenal teratomas. However, studies have shown a correlation between alpha-fetoprotein (AFP) levels and the recurrence of teratomas. This suggests that AFP can serve as a predictive index for evaluating the efficacy of treatment and the potential for a cure (20).

Although laparoscopic surgery is advantageous in terms of recovery and reduced morbidity, it has limitations, particularly in very young patients. The huge dimensions of the mass in a newborn often make open access easier and safer (21–23).

Postoperative morbidity is not uncommon and often necessitates an extended hospital stay. Reported complications include heart failure, infections, abdominal fluid collections, and chylous leakage (24). Additionally, the presence of teratomas can lead to unexpected postoperative adrenal insufficiency, particularly if the adrenal tissue is inadvertently damaged during resection (25).

This case emphasizes the importance of a multidisciplinary approach for the evaluation and management of pediatric adrenal masses. It also highlights the challenges in distinguishing between benign and malignant entities based solely on imaging and laboratory findings. Surgical intervention remains a cornerstone of management, providing both diagnostic clarity and therapeutic benefits. Further research is needed to fully understand the pathogenesis and optimal management strategies for these rare tumors.

To the best of our knowledge, there are only a limited number of reported cases in the literature regarding primary mature adrenal teratomas in pediatric patients, as summarized in (Table 1). Most of these cases were either incidentally detected or presented clinically because of abdominal distension. It is exceptionally rare for these tumors to be prenatally diagnosed. To date, only six other cases of antenatally diagnosed adrenal masses have been documented (30, 37, 44, 47, 50).

**Table 1:** Review adrenal teratoma in pediatric patients (under 18 years of age).

No.	Author	Age	Sex	Presentation	Location	Size (cm)	Gross appearance	Diagnosis
1	Engel et al. (26)	5 y	M	Asymptomatic	Right	10 × 7.5 × 3.5 cm	Cystic	MT
2	Terada et al. (27)	18 y	F	Asymptomatic	Right	15 cm	Cystic	Adrenal teratoma
3	McMillan and Horwlch (28)	17 y	M	Shortness of breath, right sided chest pain, left loin pain and weight loss	Left	8 cm	NA	Undifferenti-ated malignant teratoma
4	Lam and Lo (13)	18 y	F	Back pain	Left	11 × 8 × 7 cm	Solid	MT
5		17 y	M	Back pain	Right	7.5 × 6 × 3 cm	Cystic	MT
6	Castillo et al. (29)	8 y	M	Back pain	Right	10 cm	Cystic	MT
7	Oguzkurt et al. (30)	45 d	M	Antenatally diagnosed with suprarenal mass	Left	5.5 × 4.5 × 3 cm	Cystic + Solid	MT
8	Gow et al. (7)	9 m	M	Abdominal distension	Right	12.6 × 6.9 × 8.7 cm	Cystic	MT
9	Ersoz et al. (31)	8 y	M	Abdominal pain	Right	10 × 8.5 × 6 cm	Cystic	MT
10	Li et al. (32)	4 y	F	Asymptomatic	Left	3 × 3 cm	Cystic	MT
11	Ciftic et al. (3)	3 m	F	Vomiting	Left	14 × 10 × 8 cm	Cystic	MT
12	Yasui et al. (33)	4 m	M	Asymptomatic	Right	2.1 × 2 × 1.9 cm	Cystic + Solid	MT
13	Garg et al. (6)	2 m	F	Antenatally diagnosed with suprarenal mass	Left	7.6 × 7 × 6.5 cm	Cystic	MT
14	Peer et al. (34)	17 m	F	Abdominal distension	Right	8.7 × 4.8 × 5 cm	Cystic	MT
15	Mahzouni et al. (35)	14 y	F	Loss of appetite, fatigue, and right flank pain.	Right	8 × 8 × 6 cm	Cystic	MT
16	Narla et al. (36)	2 y	F	Abdominal pain	Right	6 × 5 × 3 cm	Cystic + Solid	MT with Carcinoid tumor
17	Garg et al. (6)	3 m	F	Abdominal distension	Right	10 × 10 × 8 cm	Cystic	MT
18	Singh et al. (37)	1 y	F	Antenatally diagnosed with suprarenal mass	Right	8.2 × 4.9 × 5.8 cm	Cystic	MT
19	Pandit et al. (38)	16 y	F	Abdominal distension	Left	12 × 10 cm	Cystic + Solid	MT
20	Baila et al. (39)	11 y	M	Back pain	Right	7 × 5 × 5 cm	Cystic + Solid	MT
21	Xu et al. (16)	2 y	M	Abdominal pain + Bloody diarrhea	Bilateral	Right: 8.5 × 8 × 6 Left: 1.6 × 1.4 × 1.3	Cystic + Solid	MT
22	Sharma et al. (40)	7 m	F	Abdominal distension	Left	9.8 × 9 × 8 cm	Cystic + Solid	MT
23	Rasul et al. (41)	7 y	F	Abdominal pain	Right	10 × 10 cm	Cystic + Solid	MT
24	Zhong et al. (19)	18 y	F	Abdominal pain	Right	8.8 cm	Cystic	MT
25	El Haddad et al. (1)	1 y	F	Abdominal distension	Left	12 × 10.9 × 8.5 cm	Cystic	MT
26	He et al. (42)	17 y	F	Asymptomatic	Right	7 × 2.5 × 2 cm	Cystic + Solid	MT
27	Banthia et al. (43)	4 y	F	Abdominal distension	Left	11 × 11.5 × 13 cm	Cystic	MT
28	Pace et al. (44)	7 d	M	Antenatally diagnosed with suprarenal mass	Right	5.6 × 5.9 × 9 cm	Cystic	MT
29	Rey-Rodriguez et al. (45)	1.3 y	F	Abdominal distension	Left	12.5 × 10.6 cm	Cystic + Solid	MT
30	Ray et al. (46)	6 m	F	Abdominal distension	Right	5 cm	Cystic + Solid	MT
31	Garcia et al. (47)	1 y	M	Antenatally diagnosed with suprarenal mass	Left	2 × 2.2 cm	Cystic	MT
32	Zhang et al. (48)	3 m	NA	Antenatally diagnosed with suprarenal mass	Right	8 × 7 × 6 cm	Cystic + Solid	MT
33	Alahdal et al. (49)	1 y	F	Abdominal distention, feeding intolerance, and constipation	Right	12 × 10 × 6.5 cm	Cystic + Solid	MT
34	Current case	18 d	F	Antenatally diagnosed with suprarenal mass	Left	8.6 × 6.5 cm	Cystic + Solid	MT

y, years; m, months; d, days; M, male; F, female; MT, mature teratoma; NA, not available.

## Conclusion

Primary adrenal teratomas in infants are exceptionally rare tumors. This case report highlights the importance of early detection, accurate diagnosis, and appropriate surgical management to achieve favorable outcomes. The comprehensive documentation of this unique case adds to the limited literature on these elusive tumors and provides valuable insights that can inform future clinical decision-making. Increased awareness and further research are needed to better understand the pathogenesis and optimize the management of these rare pediatric tumors, ultimately improving the care and outcomes of affected patients.

## Data Availability

The original contributions presented in the study are included in the article/Supplementary Material, further inquiries can be directed to the corresponding authors.
